# Music Training Positively Influences the Preattentive Perception of Voice Onset Time in Children with Dyslexia: A Longitudinal Study

**DOI:** 10.3390/brainsci9040091

**Published:** 2019-04-21

**Authors:** Aline Frey, Clément François, Julie Chobert, Jean-Luc Velay, Michel Habib, Mireille Besson

**Affiliations:** 1 ESPE de l’académie de Créteil, Université Paris-Est Créteil, Laboratoire CHArt, 94380 Bonneuil-sur-Marne, France; 2 Laboratoire Parole et Langage, CNRS et Aix Marseille Université, 13640 Aix-en-Provence, France; fclement24@hotmail.com; 3 Cognition and Brain Plasticity Group, IDIBELL, University of Barcelona, 08193 Barcelona, Spain; 4 Laboratoire de Neurosciences Cognitives, CNRS et Aix-Marseille Université, 13331 Marseille, France; julie@chobert.fr (J.C.); jean-luc.velay@univ-amu.fr (J.-L.V.); mireille.besson@univ-amu.fr (M.B.); 5 Département de Neurologie Pédiatrique, CHU Timone, 13005 Marseille, France; michel.habib@resodys.org; 6 Cuban Neuroscience Center, La Havane 4850, Cuba

**Keywords:** Music training, longitudinal study, children with dyslexia, Mismatch Negativity (MMN), syllables

## Abstract

Previous results showed a positive influence of music training on linguistic abilities at both attentive and preattentive levels. Here, we investigate whether six months of active music training is more efficient than painting training to improve the preattentive processing of phonological parameters based on durations that are often impaired in children with developmental dyslexia (DD). Results were also compared to a control group of Typically Developing (TD) children matched on reading age. We used a Test–Training–Retest procedure and analysed the Mismatch Negativity (MMN) and the N1 and N250 components of the Event-Related Potentials to syllables that differed in Voice Onset Time (VOT), vowel duration, and vowel frequency. Results were clear-cut in showing a normalization of the preattentive processing of VOT in children with DD after music training but not after painting training. They also revealed increased N250 amplitude to duration deviant stimuli in children with DD after music but not painting training, and no training effect on the preattentive processing of frequency. These findings are discussed in view of recent theories of dyslexia pointing to deficits in processing the temporal structure of speech. They clearly encourage the use of active music training for the rehabilitation of children with language impairments.

## 1. Introduction

Developmental Dyslexia (DD) is a neurodevelopmental disorder that impairs the acquisition of reading despite conventional instruction and sociocultural opportunities, normal intelligence, and motivation [1,2,3]. This disorder affects ~5% of children in primary school [1,4,5,6]. While recent computational models provide evidence that the causes of DD are likely to be multifactorial [7], deficits in phonological processing have long been considered as one of the hallmarks of DD in a large majority of dyslexic children [8,9,10]. In fact, deficient phonological processing may impact reading, writing and the acquisition of novel phonological forms [11,12,13] even with intact phonemic representations [14,15]. Specifically, the poor development of reading skills in children at risk for or with dyslexia [16,17,18] possibly reflects processing deficits of temporal and spectral acoustic cues [19,20]. In a previous experiment comparing children with dyslexia and Typically Developing (TD) children [21], we tested for this hypothesis at the preattentive level, that is, when children are not asked to focus their attention on the stimuli of interest. Therefore, these stimuli are processed implicitly rather than explicitly. We used the Mismatch Negativity (MMN), which is considered as a good index of preattentive processing ([22]; for review, see [23]). The MMN is elicited by infrequent changes in an auditory stimulus sequence of standard repeated stimuli, even when participants are watching a silent movie and not paying attention to the stimuli. The MMN, measured as the difference between the Event-Related Potentials (ERPs) to the deviant and the ERPs to the standard stimuli, can be recorded from adults as well as from children, infants, and patients which make the MMN an invaluable tool to study perceptive and cognitive processes from populations that are often difficult to test [24]. Chobert et al. [21] used a multifeature MMN design that allowed them to test, within the same auditory sequence, the preattentive processing of several types of deviant syllables that differed from the standard stimulus (/ba/) in one specific feature [25]. The multifeature MMN design is particularly useful with clinical populations that cannot be tested in long duration sessions. Syllables differed in Voice Onset Time (VOT), defined as the time interval between noise bursts at consonant release and the onset of vocal cord vibrations [26], that allows perceiving stop consonant as voiced (e.g., /b/, negative VOT ~−100 ms in French) or voiceless (e.g., /p/, VOT values higher than 0 ms in French, [27]). They also differed in vowel duration and in vowel frequency. In line with several results in the literature, we found abnormal processing of VOT and duration, but not of frequency changes in children with DD compared to typically developing (TD) children ([16,28,29,30], see [31], for abnormal processing of frequency deviant stimuli in adults with DD, and [32] for pitch discrimination deficits in sentence contexts). 

Several findings point to atypical processing of the temporal structure of speech in children with DD, particularly rise time and slow amplitude modulations of the speech envelope [19,20,33,34]. This led Goswami and collaborators to propose the temporal sampling theory of dyslexia [35,36]. They assumed that these deficits are possibly linked to abnormal synchronization of brain oscillations, at frequencies involved in syllabic and prosodic perception (theta—from 4 to 10 Hz—and delta—from 1 to 4 Hz—respectively; [35,37,38]. Very recently, Cantiania and collaborators [39] used a multifeature MMN design with nonverbal (complex tones) frequency and duration deviant stimuli. In line with the temporal sampling theory, they reported reduced left gamma power in Italian 6-month-old infants at risk for language and learning impairments compared to children with no known family deficits (see [40] for enhanced phase locking at 4–7 Hz in children with DD).

Despite evidence showing abnormal phonological processing of the structure of speech in children with DD ([21,35,41,42,43], see [29] for review), whether this deficit is causally linked to difficulties in the perception of acoustic-phonetic features still remains an open issue [21,29,35,44,45,46,47,48]. To address this causality issue, we used a longitudinal approach with children with dyslexia trained with music or with painting. The reasons for using music training in the experimental group are detailed below. Painting training was used in the control group because painting is an activity that can be as interesting and motivating for the children, thereby controlling for general factors known to influence learning such as attention and motivation.

Many results in the literature have shown that musicians are more sensitive than nonmusicians to acoustic cues that are common to music and speech sounds (i.e., duration, frequency, intensity, and timbre), possibly because processing these cues in speech and nonspeech sounds draw upon the same pool of neural resources and rely on common processing [49,50,51,52,53,54,55,56]. At the behavioral level, music training increases pitch and duration discrimination accuracy for pure and harmonic tones [57,58,59,60] and decreases discrimination thresholds for syllables that vary on temporal cues (i.e., vowel duration, VOT, and rise time; [61,62,63]). More generally, results of an increasing number of experiments demonstrated music to language transfer effects, so that music training and musical expertise influence several levels of language processing, including the processing of linguistic and emotional prosody, categorical perception, word segmentation as well as syntactic and semantic processing (for reviews see [51,64]). Taken together, these results opened the interesting perspective to use music training as a rehabilitation tool for adults and children with language deficits.

At the electrophysiological level, previous results in adults demonstrated larger amplitude and/or shorter Mismatch Negativitys (MMNs) latency in musicians than in nonmusicians for frequency, duration and timber manipulations in pure or harmonic tones (e.g., [65,66]) and in speech or speech-like stimuli (e.g., [61]). Moreover, children with high musical aptitudes and pronunciation skills showed enhanced MMNs to speech duration deviant stimuli compared to children who lacked these skills [67]. Also, using a multifeature MMN design [25], we found larger MMNs to duration deviant stimuli in 9-year-old TD children with four years of music training than in TD children with no formal music training (cross-sectional study, [68]). Moreover, the deviance size effects (i.e., the difference between large and small deviant stimuli) for VOT deviant stimuli was also larger in TD children with music training than without (Group by Deviance size interaction). Finally, as in previous studies with children with DD [16,21,29,30], no group by deviant size interaction was found for frequency changes. Taken together, these results suggest that in both adults and in TD children, music training improves several aspects of speech perception, in particular VOT and duration, possibly because increased sensitivity to features that are common to music and speech allows musicians and musically trained children to construct more reliable phonological representations of speech sounds than nonmusicians [47,49,51,52,56]. This interpretation is also in line with results showing positive correlations between musical aptitude and phonological abilities (e.g., [47,69,70,71]).

Correlation is not causality, however [72,73]. Because the studies described above used cross-sectional designs, it is not possible to ascertain that music training is at the origin of the improvements in speech perception. To directly test for causality, Chobert and collaborators [74] implemented a longitudinal study over two school years, in which TD children without formal musical background, were pseudo-randomly assigned to music or to painting training programs in a controlled, randomized trial (CRT). In line with the predictions directly issued from the results of the cross-sectional study described above [68], results for TD children showed that the MMNs to duration and VOT deviant stimuli was enhanced after 12 months of music training compared to before training but not after 12 months of painting training. No training effect was found for frequency deviant stimuli in either group. By controlling for preexisting between-group differences before training and by using pseudo-random assignment to music or painting training in a CRT, the results of Chobert et al. [74] demonstrated that enhanced sensitivity to temporal (duration) and phonological (VOT) features of syllables in TD children trained with music did not result from predispositions for music but was causally linked to music training. 

In the present study, we report results for children with DD who were involved in the same CRT as the TD children of Chobert et al. [74] and who were trained with music or painting. The multifeature MMN design [25] included the syllable “Ba” as the standard stimulus as well as large and small changes in VOT, vowel duration, and vowel frequency as deviant stimuli. The logic of the experiment is based on the results described above that were obtained with different groups of children. Compared to TD children, children with DD showed deficits in the preattentive processing of VOT and duration deviant stimuli [21]. By contrast, TD children with four years of music training (cross-sectional study, [68]) and nonmusician TD children trained with music for 12 months (longitudinal study, [74]) showed enhanced sensitivity to VOT and duration deviant stimuli compared to TD children with no formal music training or to TD nonmusician children trained with painting. Based on these results, the first aim of the present experiment was to determine whether children with DD would develop an enhanced sensitivity to VOT and to duration deviant stimuli after music training but not after painting training. Specifically, comparing children with DD involved in the two types of training, we expected the deviance size effect for VOT and duration deviant stimuli not to be significant before training (as in [21]), but to be significant after music training and not after painting training [74]. Finally, we also compared results for children with DD after music or painting training with results for TD children before training. We predicted that the deviance size effects for VOT and duration would be similar for children with DD after music training and for TD children before training (normalization of the deviance effect, no significant Group × Deviance size effect). By contrast, the deviance size effect would still be significantly different for children with DD after painting training and for TD children before training (significant Group × Deviance size interaction). 

The second aim of the present experiment was to better understand the relationship between the MMNs and the ERP components of interest. To this aim, we analysed the N100 component, an exogenous component and obligatory brain response elicited by the presentation of any stimulus, be it a sound, a light, a touch, etc. that is typically taken to reflect perceptual processing (e.g., [75]). The N100 component shows maximum amplitude (peak) at ~100 ms, and mean amplitude of the N100 is measured in a latency window surrounding the peak. Interestingly, in the studies mentioned above, results pointed to differences between children with dyslexia and TD children in the frontocentral N1 associated to stimuli with different rise times [19,20]. The N250 component probably belongs to the N200 family of components, taken to reflect stimulus categorization [76] with larger amplitude in explicit than in implicit categorization tasks [77]. The N250 is measured in the same way as the N100 component (mean amplitude in a latency window centered on the peak). In children, the N250 possibly reflects the building-up of sound representations in sensory memory [78]. We predicted enhanced amplitude of the N100 component to large, and possibly small, VOT deviant stimuli compared to standard stimuli after music training but not after painting training. Similarly, we expected the N250 component to duration deviant stimuli to be larger compared to standard stimuli after music but not after painting training. 

## 2. Materials and Methods

### 2.1. Participants

A total of 57 children participated in the study with 33 children with DD and 24 TD children attending the 3rd grade in two schools in Aix-en-Provence and Marseille. Dyslexic children in each school had been formally diagnosed with dyslexia by an interdisciplinary team of neurologists, neuropsychologists and speech therapists and they were part of a specialized dyslexia class (called CLIS in French for “CLasse pour l’Inclusion Scolaire” or class for inclusive schooling). Children in the present study were tested before training using several cognitive and reading measures (see below) in order to compare cognitive functioning and reading abilities between children with DD and TD before and after training, but not for redoing a formal diagnosis of dyslexia. Eleven children were excluded from the dyslexic group either because they left during the school year (4 children) or because of too many artifacts in their electrophysiological recordings (7 children). Out of the 22 remaining children with DD, 11 were trained with music (3 girls; 8 right-handers) and 11 were trained with painting (4 girls; 9 right-handers). Finally, three children were excluded from the group of TD children because of too many artifacts in EEG recordings and the final group comprised 21 TD children (11 girls; 18 right-handers). 

The mean chronological age at the start of the study was not significantly different in the two groups of children with DD (music group: 10.24-year-old (sd = 0.93) and painting group 10.75-year-old (sd = 0.73)), but TD children were significantly younger (8.26-year-old (sd = 0.15) than children with DD; see Table 1). Reading age was assessed with the Alouette reading test [79], which is the most commonly used standardized reading test in France, with the most reliable norms for calculating reading age [80]. Reading age of children with dyslexia in the music group was 8.14 years (sd = 0.66) and in the painting group 7.85 years (sd = 0.55), which corresponds approximately to a reading delay of 3 years. Thus, we can safely assume that children with DD who participated in the present experiment were still quite severely impaired at the time of the study. TD children were matched for reading age based on the Alouette standardized reading test. Their reading age was 8.07 years (sd = 0.33), which was not significantly different from that of the children with dyslexia (see Table 1). 

Children were tested using several tests from the Wechsler Intelligence Scale for Children (WISC-IV; Digit Span: direct and reverse, similarities, symbols; [81]), from the NEPSY battery (visual attention, auditory attention and associated responses, orientation, and visuomotor precision; [82]), from the ODEDYS battery (reading regular and irregular words as well as pseudo-words, Rapid Automatized Naming (RAN), and Phonological awareness: phoneme deletion, phoneme fusion, and nonword repetition), and the Progressive Matrices (PM47, nonverbal cognitive abilities, [83]). Results at these tests together with age, school level, gender, and socioeconomic background were used to pseudo-randomly assign children to the music or painting groups and to ensure that no significant differences existed between the two groups before training. These measures are presented in Table 1. Note that children with DD in the Dys-Mus and Dys-Paint groups showed higher scores for verbal and nonverbal IQ compared to TD children, most likely because they were almost two years older on average.

All children were native French speakers and had normal or corrected-to-normal vision, normal audition, and no known neurological deficits as determined from a detailed questionnaire completed by the parents prior to the experiment. Children had similar socioeconomic backgrounds ranging from middle to low social class as determined from the parents’ profession according to the criteria of the National Institute of Statistics and Economic Studies. Most children were involved in extracurricular activities (i.e., mainly sports), but none of the children or their parents had formal training in music or painting. 

This study was conducted in accordance with norms and guidelines for the protection of human subjects. Informed consent from the inspector and school directors as well as from the children and their parents was granted before the start of the project that was approved by the National Ethics Committee for Biomedical Research (RCB: 2011-A00172-39). Parents were informed in detail on the procedure (see below *2.2 Longitudinal Study: Procedure*) and on music and painting training that were described as challenging, interesting and rewarding experiences for their children. At the end of each school year, children from the painting group displayed their artwork at a school exhibition and children from the music group performed a concert. Children were given gifts at the end of each testing session to thank them for their participation. 

### 2.2. Longitudinal Study: Procedure

Children were tested before training and after six months of music or painting training (while children were enrolled in this program for two school years (12 months), too many children with DD had left the program after two years to obtain reliable results). In both cases, they were tested individually in a quiet room of their school in two separate sessions that included neuropsychological assessments (as described above in *2.1. Participants*) and electrophysiological tests (as described below in *2.3.MMN Experiment: Procedure*). Each session lasted for two hours (including many pauses) and was separated by four or five days. 

Two teachers professionally trained in music or visual arts were specifically hired for this project. Training lasted for 6 months (20 weeks excluding holidays) twice a week for 45 min which amounts to a total of 300 h of training. Music training was based on a combination of Kodály and Orff methods (http://www.iks.hu/; http://www.orff.de/en.html). During the music training sessions, children progressively learned how to play musical pieces of increasing complexity on diverse musical instruments including drums, timbales, guitars, and xylophones. Each session started with relaxation and vocal exercises that were followed by vocal and body games focusing on pitch, musical intervals and rhythms (finding the beat, following the beat, counting the pulse, performing polyrhythmic pieces, singing together, in canon, etc.). These games encouraged the mapping of vocal pitch modulations to hand movements. Specific time slots focused on improvising melodies and rhythms in order to foster group listening and coordination (stopping all the instruments except one, playing crescendo and decrescendo, silencing all the instruments, starting all the instruments together, starting one by one, etc.). Children also learned to synchronize their walk on the pulse while tapping in their hands (i) on the beat, (ii) on the strong beats only (2 and 4 beats), and (iii) on ¼ notes. Each session involved the recordings of short live performances in such a way that children always listened to their own productions and could comment on their quality, in order to increase their conscious awareness of the vocal and instrumental performances.

Painting training was based on the approach developed by Arno Stern (http://www.arnostern.com/) and based on the idea that children’ personal development is linked to social experiences. Autonomy and creativity are developed through painting considered as a game that is to be played together. Training sessions were built to progressively develop a better understanding of concepts such as lines and perspectives, static and dynamic figures, matter and texture, lights and colours. Children were sensitized to these different components in different training sessions using concrete approaches (e.g., combine colours that were available from cans of paint presented on a rack in the middle of the room; draw lines and perspectives; mix different textures; etc.) and different themes (e.g., favourite animals, houses and cities, nature, flowers, trees, etc.). As children were painting on large paper sheets fixed on mural panels, they learned to coordinate their movements to produce large as well as small motives in the paintings. Members of the research group coordinated the training activities and ensured that both groups of children were similarly motivated and stimulated.

### 2.3. MMN Experiment: Procedure

Children sat in a comfortable chair 1 meter from a computer screen and EEG was recorded before and after training while the children watched a silent subtitled movie displayed on a computer screen. Children were told to watch the movie without paying attention to the sounds that were presented through headphones. VOT, duration, and frequency deviant stimuli, each with two levels of deviance-size (large and small distance from the standard) were randomly presented within the auditory sequence with a sound onset asynchrony of 600 ms synchronized with vowel onset. A total of 1200 stimuli were presented with 432 deviant stimuli (72 stimuli (6% probability) for each of the 6 deviant types). All stimuli were presented within a single block that lasted for 12.2 min. At the end of the experiment, children were asked questions to ensure they had paid attention to the movie. 

### 2.4. Stimuli

**Figure 1:**
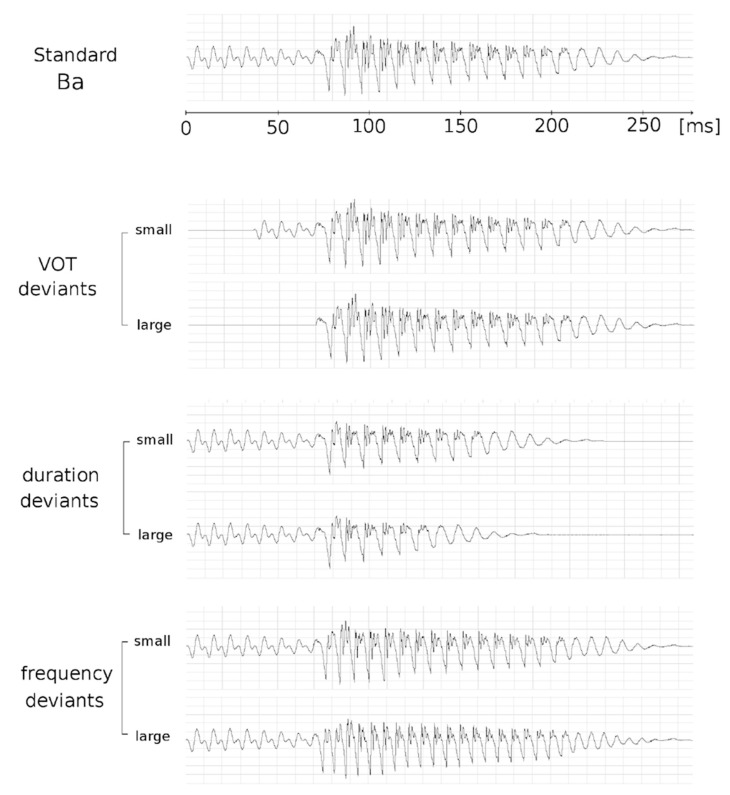
Illustration of the stimuli used in the experiment. Standard stimulus /Ba/ (Voice Onset Time (VOT): −70 ms, vowel duration: 208 ms, total duration: 278 ms and F0: 103 Hz); VOT deviant stimuli (vowel duration and F0: same as for /Ba/ but VOT for large deviant stimulus = 0 ms (/Ba_0 ms_/_)_ and VOT for small deviant stimulus = −40 ms (was /Ba_−40 ms_/); duration deviant simuli (VOT and F0: same as for /Ba/ but vowel duration for the large deviant stimulus = 128 ms and for the small deviant stimulus = 158 ms; and frequency deviant stimuli (VOT and vowel duration: same as for /Ba/ but F0 for the large deviant stimulus = 154 Hz and for the small deviant stimulus = 117 Hz).

Stimuli were syllables with Consonant-Vowel (CV) structure (see Figure 1). The standard stimulus /Ba/ was naturally produced and had a VOT of −70 ms and vowel duration of 208 ms, for a total duration of the stimulus equal to 278 ms and a fundamental frequency (F0) of 103 Hz. For VOT deviant stimuli, F0 and vowel duration were the same as for the standard stimulus but VOT changed. Large and small deviant stimuli were selected on a “Ba-Pa” continuum that comprised 9 sounds. The large deviant stimulus was “Ba_0 ms_” (VOT = 0 ms; i.e., 70 ms shorter than the standard, 100% decrease) and the small deviant stimulus was “Ba_-40 m_s” (VOT = −40 ms; i.e., 30 ms shorter than the standard, 42% decrease). For duration deviant simuli, VOT and F0 were the same as for the standard stimulus but vowel duration was shortened using “Adobe Audition” software [84]. Vowel duration was 128 ms for the large deviant stimulus (i.e., 80 ms shorter than the standard, 38% decrease; total duration large deviant = 198 ms) and 158 ms for the small deviant stimulus (i.e., 50 ms shorter than the standard, 24% decrease; total duration small deviant = 228 ms). For frequency deviant stimuli, VOT and vowel duration were the same as for the standard stimulus but the F0 of the vowel was increased using the Praat software [85]. For the large deviant stimulus, the F0 was increased to 154 Hz (i.e., 51 Hz higher than standard, 49% increase) and for the small deviant stimulus to 117 Hz (i.e., 14 Hz higher than standard, 13% increase).

### 2.5. ERP Recording and Processing

The Electroencephalogram (EEG) was continuously recorded at a sampling rate of 512 Hz with a 0–102.4 Hz band-pass using a Biosemi amplifier system (Amsterdam, BioSemi Active 2) from 32 active Ag–Cl electrodes mounted on a child-sized elastic cap (Biosemi Pintype) at standard positions of the International 10/20 System [86]. Data were re-referenced offline both to a nose reference, to verify the typical MMN inversion between Fz/Cz and the mastoids electrodes [23] and to the averaged activity over the left and right mastoids, to quantify MMN amplitude since these averages typically show a better signal-to-noise ratio than the nose-referenced averages [24,87]. EEG data were filtered with a bandpass of 1 to 30 Hz (12 dB/oct; as recommended by [24]).

The electrooculogram (EOG) was recorded from flat-type active electrodes placed 1 cm to the left and right of the external canthi, and from an electrode beneath the right eye. Three additional electrodes were placed on the left and right mastoids and on the nose. EEG data were analysed using the Brain Vision Analyser software (Version 01/04/2002; Brain Products, Gmbh). Recordings were segmented into 700 ms epochs (from −100 ms until 600 ms poststimulus onset). Epochs with electric activity exceeding baseline activity by 60 µV were considered as artifacts and were automatically rejected from further processing (~10%).

### 2.6. Data Analysis

Data from the various psychometric tests were analysed using repeated measures Multivariate Analyses of Variance (MANOVAs) that included Group (DysMus vs. DysPaint vs. TD) as a between-subjects factor, Session (before vs. after training), and Tests as within-subject factors. 

Electrophysiological data were analysed using BrainVision Analyzer v.2.0 software (Brain Products, Germany). ERPs to standard, large and small deviant stimuli were computed separately for each dimension (VOT, duration, and frequency). For VOT, the mean amplitude of the N1 component was measured in the 50 to 150 ms latency band and for duration, the mean amplitude of the N250 component was measured in the 200 to 350 ms latency band. MMNs were obtained for each deviant stimulus by subtracting ERPs to standard stimuli from ERPs to large or small deviant stimuli, separately for each participant and for each dimension (VOT, duration, and frequency) at each electrode. MMN mean amplitude was computed for each deviant over 50 ms windows (VOT: 80–130 ms, duration: 280–330 ms and frequency: 280–330 ms). Time windows were chosen based on visual inspection and on results of previous studies analysing MMN in children [21,74]. 

Repeated-measures of ANalysis Of VAriance (ANOVAs) were computed on MMN mean amplitude, as well as on N100 and N250 mean amplitude, for each dimension separately (VOT, duration, and frequency). Analyses typically included Group (DysMus vs. DysPaint) as a between-subject factor, and Session (before vs. after training), deviance size for MMNs analyses (large vs. small deviant stimuli), Anterior–Posterior Dimension (frontal, central, and parietal) and Laterality (left, central, and right) as within-subject factors, or only frontal sites (F3, Fz, and F4). Separate ANOVAs were conducted for each session separately when results of interactions including the Group and Session factors were significant. Finally, further analyses were also conducted to test for the hypothesis of a normalization of VOT and duration processing with music training that included the group of TD children (before training) and the groups of children with DD after music or painting training. Greenhouse–Geisser corrections were applied when appropriate and conservative post hoc Tukey tests (reducing the probability of Type I errors) were used to determine the source of significant interactions.

## 3. Results

### 3.1. Neuropsychological and Speech Assessments

No between-group differences were found before training (F (1,20) = 2.04; *p* = 0.17). Overall, the level of performance was higher after six months of training than before training (main effect of Session: (F (1,20) = 11.6; *p* < 0.003, see Table 2 for the main effect of Session in each specific test). This improvement was not significantly different in the music and painting training groups (main effect of Group: F < 1; Group by Session interaction F < 1).

### 3.2. MMN Amplitude

**Figure 2:**
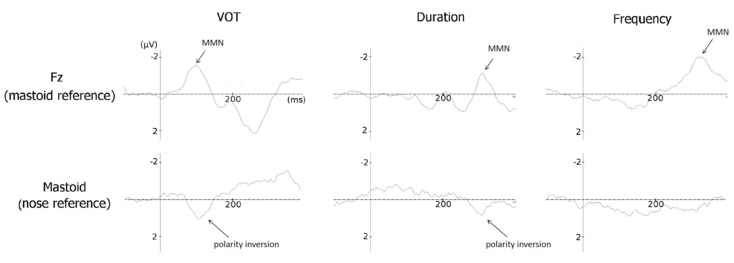
Mismatch Negativities (MMNs—i.e., Event-Related Potentials (ERPs) to the deviant stimuli minus ERPs to standard stimuli) averaged across large and small deviant stimuli and before and after training at Fz (with mastoid reference, top) and at mastoid electrode (mean of left and right mastoid electrodes, with nose reference, bottom) for Voice Onset Time (VOT, left), Duration (middle), and Frequency (right) deviant stimuli. Note the clear polarity inversion between Fz and mastoid for VOT and duration changes but not for frequency changes.

As is typical in MMN paradigms, MMNs to deviant stimuli in VOT, duration, and frequency in children with dyslexia showed the typical polarity inversion between Fz and the mastoids electrodes when using the nose reference (see Figure 2). 

Moreover, independently of the type of deviant stimuli, MMNs were always larger over fronto-central regions than over parietal regions (main effect of Anteroposterior factor for VOT: F(2,40) = 11.22, *p* < 0.001; for duration: F(2,40) = 7.81, *p* < 0.001; and for frequency: F(2,40) = 18.40, *p* < 0.001; see Table 3). Based on these results, further analyses were conducted on the averaged responses for each deviant over frontal sites (F3, Fz, and F4). Analyses of the deviance size effect for each type of deviant stimuli (VOT, duration, and frequency) are reported below.

#### 3.2.1. VOT (MMN Amplitude)

Results of ANOVAs on MMN amplitude including Group (DysMus vs. DysPaint) as a between-subject factor as well as Session (before vs. after training), Deviance size (large vs. small VOT deviant stimuli) and Laterality (F3 vs. Fz vs. F4) as within-subjects factors showed that the main effects of Group and Session were not significant (both Fs < 1) but the Group × Session × Deviance size was significant (F(1,20) = 3.39, *p* < 0.04).

**Figure 3:**
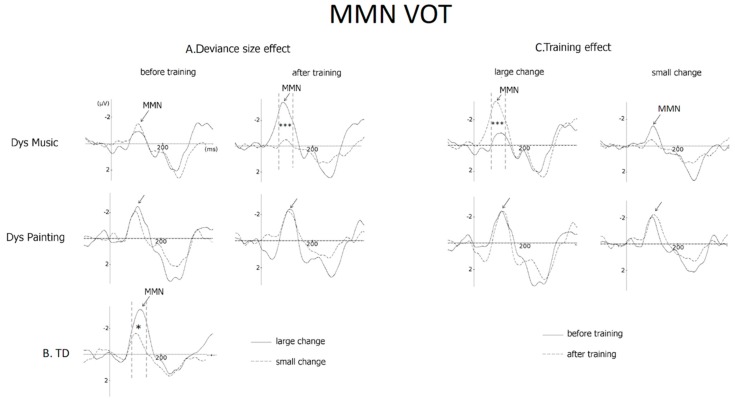
Mismatch Negativities (MMNs) to Voice Onset Time (VOT) deviant stimuli at Fz. (**A**) The deviance size effect (i.e., the difference in MMNs mean amplitude between large and small deviant stimuli) is illustrated before and after training for children with dyslexia trained with music (DysMus, top) or with painting (DysPaint), middle) and (**B**) for Typically Developing children (TD). (**C**) The training effect to large and small changes in VOT is illustrated before (solid lines) and after training (dashed line) for children with dyslexia in the music and in the painting groups.

Results of separate ANOVAs before training revealed that the VOT deviance size effect on MMN amplitude was not significant either in DysMus (large = −1.21 µV and small = −1.27 µV, *p* < 0.99) or in DysPaint (large = −2.07 µV and small = −1.80 µV, *p* < 0.99; Group × Deviance size: F < 1). By contrast, after training, the deviance size effect was significant in DysMus (*p* < 0.003), with larger MMNs to large (−2.48 µV) than to small (−0.42 µV) VOT changes, but not in DysPaint (large = −1.99 µV and small = −1.69 µV, *p* = 0.93; Group × Deviance size: (F(1,20) = 6.03, *p* < 0.02; see Figure 3A). In other words, in DysMus, MMNs to large VOT deviant stimuli were larger after (−2.48 μV) than before training (−1.21 μV, *p* < 0.001) with no training-related differences in DysPaint (before training: −2.07 µV and after training: −1.99, µV, *p* < 0.99). For small VOT changes, the differences between after and before training did not reach significance either for DysMus or for DysPaint (see Figure 3C). 

As mentioned in the introduction, we hypothesized that the deviance size effects for VOT and duration would be similar for children with DD after music training and for TD children before training. In other words, we expected a normalization of the deviance effect in the music group but not in the painting group. To specifically test for this hypothesis, we compared the group of TD children (before training) and DysMus and DysPaint groups after training. Results showed that the Group by Deviance size interaction was significant (F(2,40) = 4.28, *p* < 0.02). Separate comparisons showed that the deviance size effect was similar for TD children and DysMus (Group × Deviance size: F(1,30) = 1.16, *p* < 0.30), but it was larger for TD children than for DysPaint (Group × deviance size: F(1,30) = 4.37, *p* < 0.04). As can be seen on Figure 3A,B, the deviance size effect is significant for DysMus and for TD children, but not for DysPaint.

#### 3.2.2. VOT (N1 Amplitude)

Results of ANOVAs on N1 amplitude that included Group (DysMus vs. DysPaint) as a between-subject factor as well as Session (before vs. after training), Deviance size (large vs. small VOT deviant stimuli), Anterior-Posterior Dimension (frontal, central, and parietal), and Laterality (Left, Central, and Right) as within-subject factors were very similar to those reported above for MMN amplitude. The main effects of Group and Session were not significant (F < 1 and (F(1,20) = 3.48, *p* < 0.07, respectively) but the Group × Session × Deviance size was significant (F(1,20) = 5.69, *p* < 0.03).

**Figure 4:**
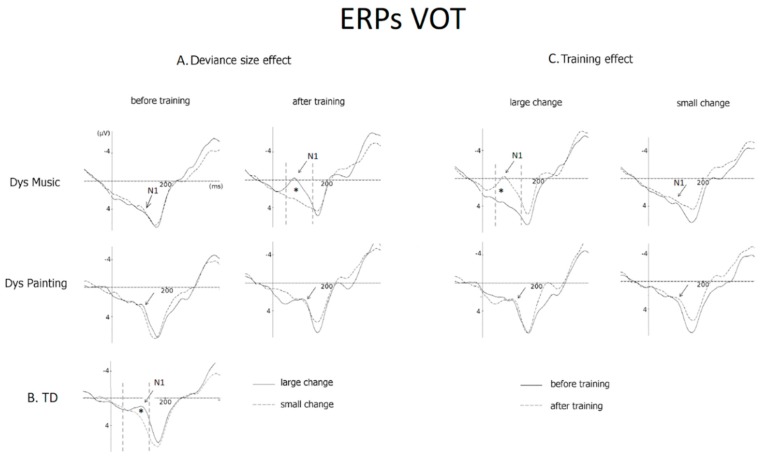
ERPs (original averages) to VOT deviant stimuli at Fz. (**A**) The deviance size effect (i.e., ERPs to large and small deviant stimuli) is illustrated before and after training for children with dyslexia trained with music (DysMus, top) or with painting (DysPaint), middle) and (**B**) for Typically Developing children (TD). (**C**) The training effect to large and small changes in VOT is illustrated before (solid lines) and after training (dashed line) for children with dyslexia in the music and in the painting groups.

Before training, results of separate ANOVAs revealed that the VOT deviance size effect on N1 amplitude was not significant either in DysMus (large = 2.12 µV and small = 1.98 µV, *p* = 0.99) or in DysPaint group (large = 1.32 µV and small = 1.54 µV, *p* = 0.96; Group by Deviance size: F< 1). By contrast, after training, the deviance size effect was significant in DysMus (*p* < 0.02) with larger N1s to large (0.83 µV) than to small (1.93µV) VOT deviant stimuli, but not in DysPaint (large = 1.93 µV and small = 1.59 µV, *p* = 0.85; Group by Deviance size: (F(1, 20) = 5.14, *p* < 0.03); see Figure 4A). 

In DysMus, the N1 to large VOT deviant stimuli was larger (i.e., less positive) after (0.83 μV) than before training (2.12 μV, *p* < 0.03) across all scalp sites, with larger differences over the midlines (*p* < 0.001) and the right hemisphere locations (*p* < 0.001) than over the left hemisphere (*p* < 0.05). No such differences were found in DysPaint (after training: 1.93 μV and before training: 1.32 μV, *p* = 0.85; Group by Session by Deviance size by Laterality: F(2,40) = 2.75, *p* < 0.03). For small VOT changes, the differences between after and before training were not significant in either group (DysMus = 1.93 µV vs. 1.98µV; DysPaint = 1.59 µV vs. 1.54µV; see Figure 4C).

To specifically test for the normalization of VOT processing with music training, we compared the group of TD children (before training) and the groups of DysMus and DysPaint after training. Results showed that the Group by Deviance size interaction was significant (F(2,40) = 7.56, *p* < 0.001). Separate comparisons showed that the deviance size effect on N1 amplitude was similar for TD children before training and DysMus after music training (Group × Deviance size: F < 1), but it was larger for TD children than for DysPaint after painting training (Group × deviance size: F(1,30) = 14.72, *p* < 0.001; compare Figure 4A,B).

### 3.3. Duration

#### 3.3.1. Duration (MMN Amplitude)

**Figure 5:**
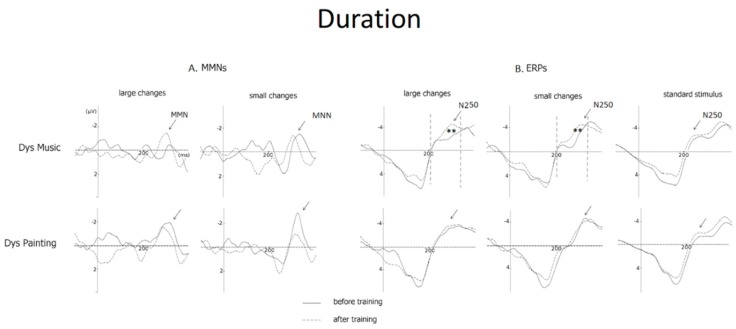
(**A**) Mismatch Negativities (MMNs) and (**B**) ERPs at Fz to large and small duration changes as well as for standard stimuli (for ERPs) before training (solid line) and after training (dashed line) for children with dyslexia in the music group (Dys Mus) and in the painting group (DysPaint).

Results of ANOVAs on MMN amplitude including Group (DysMus vs. DysPaint) as a between-subject factor as well as Session (before vs. after training), Deviance size (large vs. small duration deviant stimuli) and Laterality (F3 vs. Fz vs. F4) as within-subjects factors showed that neither the main effects of Group and Session (F(1,20) = 1.13, *p* = 0.30 and F(1,20) = 2.54, *p* = 0.12, respectively) nor the Group by Session by Deviance size and the Group by Session by Deviance size by Laterality interactions were significant (F < 1). Thus, in contrast to our hypothesis, the deviance size effect after training was not significantly different for DysMus or DysPaint (see Figure 5A). 

#### 3.3.2. Duration (N250 Amplitude)

Analyses of the N250 amplitude revealed that the main effects of Group and Session were not significant (F < 1 and F(1,20) = 1.37, *p* < 0.30), but there was a trend toward significance in the interaction Group by Session by Deviance size by Laterality (F(4, 80) = 2.32, *p* < 0.06). 

Separate ANOVAs for DysMus showed that the N250 to both large and small duration deviant stimuli increased in amplitude from before to after training over frontal and central sites (Session by Anteroposterior interaction: F(2,20) = 5.39, *p* < 0.01; see Table 4). By contrast, for DysPaint, the N250 to duration deviant stimuli was not significantly different before and after training (main effect of Session: F < 1; see Figure 5B). 

### 3.4. Frequency (MMN and N250 Amplitude)

**Figure 6:**
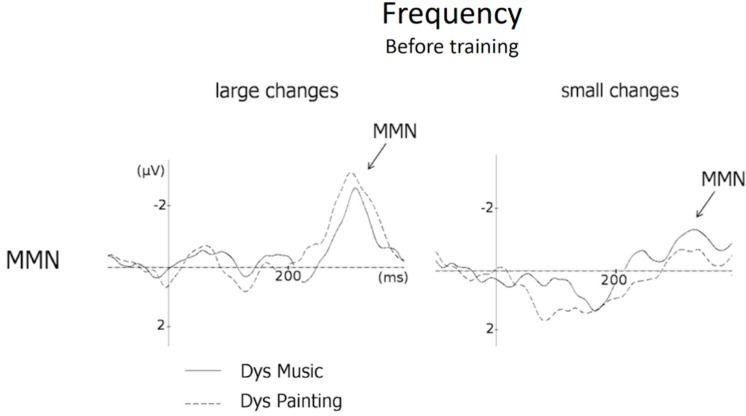
MMN at Fz to large and small frequency changes before training. MMNs are overlapped for children with dyslexia in the music group (solid line) and in the painting group (dashed line).

Results of ANOVAs showed that before training the main effect of Group was significant on MMN amplitude (F(1,20) = 3.94, *p* < 0.05, see Figure 6), with larger MMNs in DysPaint (−3.10 µV) than in DysMus (−1.54 µV), thereby precluding further analyses of training effects on the preattentive processing of frequency deviant stimuli.

## 4. Discussion

The aim of this longitudinal study was to determine whether music training improves the preattentive processing of VOT and duration deviant stimuli in children with DD and normalizes the deviance size effects so that children with dyslexia after music training would not significantly differ from TD children before training. Results clearly support this hypothesis for VOT deviant stimuli but were not as clear-cut for duration deviant stimuli, as discussed below together with results for frequency deviant stimuli.

### 4.1. VOT Deviant Stimuli

The present study capitalized on previous results in the literature and on specific findings from our group using the same design and stimuli, that showed (1) deficits in the preattentive processing of VOT deviant stimuli in children with DD compared to TD children (cross-sectional study, [21]), (2) improved processing of VOT deviant stimuli in TD children with four years of music training, on average, compared to control nonmusician TD children (cross-sectional study, [68]), and (3) improved processing of VOT deviant stimuli in nonmusician TD children trained with music for 18 months compared to TD children training with painting (longitudinal study, [74]). The novelty of the present experiment is to use a longitudinal approach as in Chobert et al. [74] but with children with DD instead of TD children. Based on the findings summarized above, we hypothesized that six months of music training, but not of painting training, would enhance the preattentive processing of VOT in children with DD. In line with these hypotheses, the VOT deviance size effect was not significant in children with DD (either DysMus or DysPaint) before training but it was significant after music training and not after painting training. This was mainly due to an increase in MMN amplitude to large VOT deviant stimuli with no significant difference for small VOT deviant stimuli. Moreover, the VOT deviance effect for DysMus after music training was not significantly different from the VOT deviance size effect in the control group of TD children before training. By contrast, the VOT deviance size effect was still significantly smaller in DysPaint after training than in TD children before training. Thus, music training, but not painting training, helped to normalize the deviance size effect for VOT deviant stimuli in children with dyslexia. Because the VOT deviance size effect was not significant in either group of children with dyslexia before training and nonsignificant in DysPaint after training, the normalization found for DysMus is more likely to result from the positive influence of music training than from the influence of general factors such as maturation, attention and/or motivation [73]. 

Results of this longitudinal CRT in children with DD showed that music training improved sensitivity to VOT, a phonological parameter that is contrastive in French, and that in the present design allowed to discriminate the deviant (/pa/) from the standard syllables (/ba/). In other words, music training improved categorical perception, a cornerstone of speech perception that has been largely investigated in children with dyslexia [88,89,90,91]. Our findings at the preattentive level, as reflected by increased MMN and N1 amplitude to VOT deviant stimuli, are in line with previous results evidencing enhanced categorical perception with music training in children with DD. For instance, Habib et al. [92] tested for the influence of a Cognitive Musical Training (CMT) method in children with DD and in TD children matched on reading age. The CMT method was designed by speech therapists to include three main components: (1) an auditory component to mobilize the language-music similarity, with exercises based on pitch, duration, tempo, pulsation, and rhythm that aimed at developing both the perception and the production sides; (2) a motor component to engage the child into rhythm production and imitation (e.g., tapping in synchrony with sounds, tapping the written notation of a rhythm…); and (3) a cross-modal component, to tap into simultaneous processing of information from different modalities including auditory, visual, sensory, and motor modalities as well as their combinations. The rhythmic aspect was always emphasised (for more details, see [93]).

CMT was used for 18 h either during three consecutive days (Experiment 1) or spread over six weeks (Experiment 2), and was based on rhythm production and imitation as well as on cross-modal integration of information from auditory, visual, sensory, and motor modalities. Results showed that CMT improved the level of performance of children with DD in both the identification (/ba/ to /pa/ continuum) and the discrimination (different /ba/ - /pa/ pairs from the continuum) tasks used to investigate categorical perception. Moreover, differences between children with DD and control TD children after CMT were no longer significant. More generally, such findings are also in line with previous reports that music training improved categorical perception of speech sounds in both younger and older musicians compared to controls without formal music training [94]. Increased auditory sensitivity may thus be one of the driving forces behind enhanced categorical perception with music training.

As mentioned above, MMN amplitude to large VOT deviant stimuli significantly increased from pre to post training but results showed no effect of music or painting training on the preattentive processing of small VOT deviant stimuli. It could be that the difference between the standard and small VOT deviant stimuli was too small to be preattentively detected or that the duration of music training was not sufficiently long to increase auditory sensitivity to small changes in VOT. In line with this interpretation, results of the longitudinal study with TD children conducted by Chobert et al. [74] showed increased MMN to small VOT deviant stimuli after 12 months of music training, but not after six months of music training as done here. Thus, for both TD children and children with DD, more than six months seem necessary to find an effect of active music training on subtle changes in VOT. However, the results of Habib et al. [92] described above showed that only 18 h of music training influenced categorical perception. These different results are possibly linked with differences in the CMT method and the type of music training used here or with the small VOT deviant stimuli being less different from the standard than the stimuli used in the Habib et al. [92] experiment.

Results for the N1 component paralleled those found for the MMN: the VOT deviance size effect on N1 amplitude was not significant before training in either group of children with DD but it was significant after music training (i.e., larger N1 to large than to small VOT changes) and not after painting training. The normalization of the VOT deviance size effect was also reflected on N1 amplitude in DysMus after training (similar to the deviance effects in TD children) but not in DysPaint. 

From an acoustic perspective, VOT deviant stimuli differed from standard stimuli right at stimulus onset. Thus, both the obligatory N1 response to stimulus onset and the MMN effects developed in the same latency band. This raises the possibility that the VOT deviance size effect reflects differences on N1 rather than on MMN amplitude [22,87,95,96]. However, two arguments lead us to consider that they are overlapping but different effects. First, the MMN and N1 deviance size effects showed a different scalp distribution. In line with previous studies (see [23,24], for reviews) the MMNs to large VOT deviant stimuli were clearly localized over frontal sites. By contrast, enhancement in N1 amplitude to large VOT deviant stimuli after music training was larger over midlines and right hemisphere than over left hemisphere sites. Thus, different generators seem at the origin of the N1 and MMN effects observed at the scalp. Second, while the N100 component is always time-locked to stimulus onset, the latency of the MMN varies as a function of when participants can perceive the deviance. Thus, in the present experiment, the MMN peaked earlier, ~100 ms, for VOT deviant stimuli than for duration and frequency deviant stimuli—~300 ms (see Figure 1, Figure 3, Figure 5, and Figure 6). Thus, in spite of the temporal overlap between the N1 and the MMN to VOT changes, these two effects may reflect the positive influence of music training on different processes: on the early perceptual processing of VOT (as reflected by the N1 component) and on the preattentive detection of a mismatch between standard and large VOT deviant stimuli (as reflected by the MMN).

Taken together, these results support the hypothesis that music training positively impacts the preattentive processing of VOT, mainly by influencing the perception of large VOT changes. Specifically, these results demonstrate that music training, but not painting training, can normalize the preattentive perception of VOT in children with dyslexia since, after music training, no significant differences were found between children with DD and TD children. These results nicely complement previous findings with TD children trained with music or painting [74]. Finally, they demonstrate transfer effects from music training to the perception of an acoustic-phonological feature in speech—VOT—which is important for categorical perception.

### 4.2. Duration Deviant Stimuli

In contrast to our hypothesis, results on MMN amplitude revealed no advantage of music training over painting training on the preattentive processing of large and small duration deviant stimuli (no Group by Session by Deviance size interaction). This conclusion is also in contrast with results reported by [67] showing enhanced MMNs to speech duration deviant stimuli in 10-to-12-year-old children with high musical aptitudes, and with results of Chobert et al. [68], showing larger MMNs to duration deviant stimuli in musician children with an average of four years of music training compared to children with no formal music training. Several factors may explain these discrepancies: the children with DD involved in the present study may not have such high musical aptitudes as in previous studies, may not be as motivated as children who choose to be involved in formal music training or the teaching methods used here are possibly different than those used in classic music school. Moreover, results of the longitudinal study over two years conducted by Chobert et al. [74] with TD children showed that the MMN to duration deviant stimuli was larger after 12 months of music training, but not after six months of music training. It is therefore not surprising that children with DD showed no significant effect of six months of music training on the preattentive processing of duration deviant stimuli. As mentioned above, these children were involved in the same two-year project as the TD children of Chobert et al. [74] but the attrition rate at the end of the second year was too high to obtain reliable results (only six dyslexics were left in the music group and four dyslexics in the painting group). 

In contrast with the MMN results, the amplitude of the N250 to both large and small duration deviant stimuli was larger after six months of music training than before training over fronto-central sites with no difference in the painting group. Thus, in line with results at the behavioral and electrophysiological levels showing that musical expertise improved duration discrimination accuracy in speech and perception of the metric structure of speech (e.g., [60,97]), music training seemed to enhance the preattentive perception of duration changes in children with dyslexia. However, this interpretation needs to be considered with caution, first because the Group by Session by Deviance size by Laterality interaction was only marginally significant (*p* < 0.06) and, second, because we found an increase in N250 amplitude that was as large for large than for small duration deviant stimuli. Thus, in contrast to our hypothesis, the deviance size effect was not significant. 

### 4.3. Frequency Deviant Stimuli

As mentioned in the methods section, children with DD were pseudo-randomly assigned to music or to painting training to ensure that no between-group differences were found before training. The assignment was based on different factors (i.e., age, school level, sex, and socioeconomic background) as well as on the results at WISC-IV [81], NEPSY [82] and ODEDYS batteries [98]. However, it was not possible to control that no between-group differences were present before training on all the electrophysiological measures of interest. When results were analysed at the end of the experiment, they showed that before training, MMNs were larger in the painting group than in the music group. As a consequence, we did not conduct further analyses as it would be extremely difficult to disentangle the differences due to training from these significant pretraining differences. 

Note that while it could have been interesting to determine whether 6 months of music training improves the preattentive processing of frequency deviant stimuli in children with DD, previous results with the same stimuli showed no significant improvements after 12 months of music training in TD children [74]. Based on these findings, we did not expect to find an effect of only six months of music training in children with DD. Moreover, as mentioned above, mixed results have been reported in cross-sectional studies comparing the processing of frequency changes in children with DD and TD children. For instance, Halliday and collaborators [40] reported no main effect of group on MMN amplitude but smaller Late Discriminative Negativity (LDN; 350–550 ms poststimulus onset) to small frequency deviant stimuli in children with DD than in TD children. Similarly, Hämäläinen and collaborators [20] reported differences between children with reading disabilities and control children in the rapid processing of pitch changes on P3a but not on MMN amplitude. By contrast, Baldeweg et al. [31] found adult dyslexics to be impaired in auditory frequency discrimination, as reflected by the MMN and Maurer and collaborators reported that children at familial risk for dyslexia have more difficulties than controls to detect frequency deviant stimuli [99].

### 4.4. Psychometric Tests

Results of MANOVAs including the various psychometric tests as well as DysMus vs. DysPaint and before vs. after training as factors revealed that the level of performance was higher after six months of training than before training on several tests, including reading (Alouette and reading irregular words), verbal IQ (similarities), Rapid Automatized Naming (RAN), phoneme fusion, and Attention (auditory attention and orientation). This main effect of Session can be explained by repetition effects: the same tests were presented twice with typically higher level of performance on second that on first presentation. It may also reflect maturation effects since children were seven months older after compared to before training. However, and most importantly, we found no significant difference between DysMus and DysPaint, so that six months of music training did not improve the attentive use of phonological representations more than painting training. These results stand in contrast with previous results in the literature showing a positive impact of music training on speech perception and on different levels of language processing, including reading abilities. For instance, significant correlations between rise time perception and reading/spelling abilities have been reported in previous studies (e.g., [35,100]). Huss et al. [43] also found strong correlations between the perception of musical meter, sound rise time, phonological awareness, and reading abilities in children with DD and in TD children. In line with the temporal sampling theory of dyslexia proposed by Goswami and collaborators [35,36], rise time is possibly more important for syllabic discrimination than VOT. Another interesting interpretation of our discrepant results is based on the type of music training necessary to find improvements in phonological awareness and reading. For instance, Flaugnacco et al. [101] conducted a CRT with children with DD very similar to the CRT conducted by Chobert et al. [74] and here. They also used music training based on a combination of Kodály and Orff methods but with a strong focus on rhythm, temporal processing, and sensorimotor synchronization. Under these conditions, the level of performance in a metric perception task (i.e., perceiving changes in note duration within recurrent series) predicted reading speed and accuracy as well as phonological processing in Italian children with DD. Thus, focusing training on the rhythmic and motor components of music is possibly more efficient to normalize the attentive perception of the temporal structure of speech in children with DD (e.g., [19]). Finally, other factors may also account for these contrasting results, such the age of the children with DD, the severity and homogeneity of the dyslexia deficit and the impact of the speech therapy.

## 5. Conclusions

The present results reveal that six months of music training clearly improved the preattentive processing of VOT—a phonological cue determinant for categorical perception [92]—as reflected by strong changes in MMN and N1 amplitude. To a lesser extent, results also showed a larger influence of music compared to painting training on the processing of vowel duration in children with DD, as reflected by increased N250 amplitude to both large and small duration deviant stimuli. However, in contrast to previous results [43,92,100,101], six months of music training were not sufficient to improve attentive phonological processing or reading abilities, as revealed by results at the standardized psychometric tests. Thus, music training differentially influenced the preattentive processing of VOT and duration, as measured with the MMN to large and small deviant stimuli (and the deviance size effect), and the attentive processing of phonological cues, as measured in the psychometric tests. It is very possible that the effects of music training are seen in the brain waves before being seen in behavior, and that they would manifest in the various behavioral tests after longer training. Moreover, based on the discussion above, focusing music training on the rhythmic and motor components of music is possibly the most efficient strategy to improve speech perception. There is already strong evidence in the literature that improving temporal processing has a strong impact on phonological and reading abilities as well as on semantic and syntactic processing. For instance, Przybylski et al. [102] reported that the level of performance in a syntactic task (decide whether a spoken sentence was syntactically correct or incorrect: e.g., “Laura has/have forgotten her violin”) was increased by the prior presentation of rhythmic primes (a succession of notes played either regularly or irregularly). There is also recent evidence for preserved semantic processing in both children [103] and adults with dyslexia [104].

Taken together, these results provide new evidence for a positive influence of music training on preattentive speech perception in children with dyslexia. Importantly, because children were pseudo-randomly assigned to music or to painting training these results more likely reflect the impact of active music training than the influence of genetic predispositions for music. The direct implication of these findings is that rehabilitation methods of dyslexia should focus, at least in part, on restoring the ability to process temporal structures that sequentially unfold in time, such as speech and music. More generally, and based on recent results pointing to multi-deficits (orthographic, phonological and vocabulary) rather than single-deficit problems in children with dyslexia [7], rehabilitation methods should aim at increasing the integration of the different components that are important for reading and learning, using music training and possibly preserved semantic processing abilities in children with dyslexia to overcome their difficulties [103].

## Figures and Tables

**Table 1:** Before training. Results of children with dyslexia (DysMus and DysPaint) and of typically developing readers (TD) who were matched on reading age, on measures of memory, on verbal and nonverbal intelligence, on phonology, on reading regular words, irregular words and pseudo-words, and on visual and auditory attention. For each test, the number in brackets (e.g., /44) corresponds to maximum score.

Test	TD	DysM.	DysP.	F(2,40)	Post hoc comparisons
**Chronological age** (month)	99.14	122.91	129.00	60.25, *p* < 0.001	DysM. vs. TD: *p* < 0.001 DysP. vs. TD: *p* < 0.001 DysM. vs. DysP.: ns
**Reading age ^a^** (month)	92.44	87.24	86.27	F < 1	
**Memory ^b^** Digit Span (/32)	13.38	11.45	11.73	3.71, *p* = 0.03	DysM. vs. TD: *p* < 0.05 DysP.vs TD: ns DysM. vs. DysP.: ns
**Verbal IQ** Similarities (/44) ^b^	15.67	17.73	18.36	F < 1	
**Nonverbal IQ** Symboles (/60) ^b^	16.38	20.73	16.82	2.16, *p* = 0.13	
**Nonverbal IQ** Progressive Matrices (/36) ^c^	26.81	28.91	27.64	F < 1	
**Phonology^d^** RAN (seconds)	28.58	27.18	29.45	F < 1	
**Phonology ^d^** Phoneme Deletion (/10)	5.58	5.54	4.82	F < 1	
**Phonology ^d^** Phoneme Fusion (/10)	5.92	5.64	5.18	F < 1	
**Phonology ^d^** Nonword repetition (/20)	17.58	16.64	17.09	F < 1	
**Reading irregular words** (/20)	7.91	4.45	6.90	2.24, *p* = 0.15	
**Reading regular words** (/20)	14.27	9.27	12.36	3.07, *p* = 0.08	
**Reading of pseudowords** (/20)	13.60	8.82	10.08	7.84, *p* < 0.03	DysM. vs. TD: *p* < 0.02 DysP. vs. TD: p < 0.05 DysM. vs. DysP.: ns
**Attention ^e^** Visual Attention Score (/45)	17.08	16.00	18.09	F < 1	
**Attention ^e^** Auditory Attention (/132)	93.92	84.36	89.27	F < 1	
**Attention ^e^** Orientation (/10)	6.56	7.45	7.18	F < 1	
**Attention ^e^** Visuomotor precision (/52)	22.42	25.36	24.09	F < 1	
**Attention ^e^** Arrows (/30)	19.33	20.73	20.09	F < 1	

^a^ Alouette Standardized Reading Test. ^b^ Wechsler Intelligence Scale for Children WISCIV. ^c^ Progressive Matrices PM47. ^d^ ODEDYS. ^e^ NEPSY.

**Table 2:** Results of children with dyslexia (DysMus and DysPaint) after 6 months of training, on measures of memory, verbal and nonverbal intelligence, phonology, reading regular and irregular words and pseudo-words, and visual and auditory attention. T0: before the start of the experiment and T6: six months after.

Test	DysM.	DysP.	Main Effect Session F(1,20)	Post hoc Comparisons
**Reading age ^a^** (month)	90.18	90.45	18.50 *p* < 0.001	T0 = 82.64 < T6 = 90.32
**Memory ^b^** Digit Span (/32)	11.73	11.18	F < 1	
**Verbal IQ** Similarities (/44) ^b^	23.55	24.09	34.93 *p* < 0.001	T0 = 18.04 < T6 = 23.82
**Nonverbal IQ** Symboles (/60) ^b^	22.64	19.00	F = 2.29 *p* < 0.15	
**Nonverbal IQ** Progressive Matrices (/36) ^c^	30.36	27.64	F < 1	
**Phonology ^d^** RAN (seconds)	23.27	23.64	5.62 *p* < 0.03	T0 = 32.44 > T6 = 26.40
**Phonology ^d^** Phoneme Deletion (/10)	6.00	5.18	F < 1	
**Phonology ^d^** Phoneme Fusion (/10)	7.27	7.09	11.20 *p* < .003	T0 = 5.41 < T6 = 7.18
**Phonology ^d^** Nonword repetition (/20)	17.27	18.00	F < 1	
**Reading irregular words** (/20)	6.82	8.45	8.52 *p* < 0.01	T0 = 5.68 < T6 = 7.64
**Reading regular words** (/20)	11.18	12.00	F < 1	
**Reading Pseudowords** (/20)	8.64	11.73	F < 1	
**Attention ^e^** Visual Attention Score (/45)	18.55	17.55	F < 1	
**Attention ^e^** Auditory Attention (/132)	106.18	98.91	5.76 *p* < 0.03	T0 = 86.82 < T6 = 102.55
**Attention ^e^** Orientation (/10)	8.18	8.18	10.87 *p* < 0.005	T0 = 7.32 < T6 = 8.18
**Attention ^e^** Visuomotor precision (/52)	26.91	26.73	F = 2.13 *p* < 0.16	
**Attention ^e^** Arrows (/30)	20.45	19.00	F < 1	

**Table 3:** Mean MMNs amplitude in children with dyslexia averaged across sessions and across large and small deviant stimuli on Voice Onset Time (VOT), duration, and frequency at Frontal, Central, and Parietal sites.

	VOT	Duration	Frequency
Frontal	−1.61 µV	−1.12 µV	−1.85 µV
Central	−1.27 µV	−1.16 µV	−1.24 µV
Parietal	−0.64 µV	−0.69 µV	−0.59 µV

**Table 4:** Mean N250 amplitude (in microvolts) at Frontal and Central sites for children with DD trained with music (DysMus) or with painting (DysPaint) before and after training, for large and small duration deviant stimuli.

		Frontal		Central	
		Before	After	Before	After
	Large Dev.	−1.96	−2.99	−1.90	−2.60
DysMus	Small Dev.	−1.88	−2.76	−1.54	−2.13
	Large Dev.	−1.44	−1.37	−1.66	−1.38
DysPaint	Small Dev.	−0.86	−1.19	−0.96	−1.51

## References

[1] Démonet J.F., Taylor M.J., Chaix Y. (2004). Developmental dyslexia. Lancet.

[2] Habib M. (2000). The neurological basis of developmental dyslexia. Brain.

[3] Collective Expertise INSERM, CNDRSDI (2007). Dyslexie, Dysorthographie, Dyscalculie: Bilan des Données Scientifiques.

[4] Norton E.S., Beach S.D., Gabrieli J.D. (2015). Neurobiology of dyslexia. Curr. Opin. Neurobiol..

[5] Snowling M.J. (2000). Dyslexia.

[6] Valdois S., Bosse M.L., Tainturier M.J. (2004). The cognitive deficits responsible for developmental dyslexia: Review of evidence for a selective visual attention disorder. Dyslexia.

[7] Perry C., Zorzi M., Ziegler J.C. (2019). Understanding Dyslexia Through Personalized Large-Scale Computational Models. Psychol. Sci..

[8] Ramus F. (2003). Developmental dyslexia: Specific phonological deficit or general sensorimotor dysfunction?. Curr. Opin. Neurobiol..

[9] Saksida A., Iannuzzi S., Bogliotti C., Chaix Y., Demonet J.F., Bricout L., Billard C., Nguyen-Morel M.A., Le Heuzey M.F., Soares-Boucaud I. (2016). Phonological skills, visual attention span, and visual stress in de- velopmental dyslexia. Dev. Psychol..

[10] White S., Milne E., Rosen S., Hansen P., Swettenham J., Frith U., Ramus F. (2006). The role of sensorimotor impairments in dyslexia: A multiple case study of dyslexic children. Dev. Sci..

[11] Ahissar M., Lubin Y., PutterKatz H., Banai K. (2006). Dyslexia and the failure to form a perceptual anchor. Nat. Neurosci..

[12] Kimppa L., Shtyrov Y., Partanen E., Kujala T. (2018). Impaired neural mechanism for online novel word acquisition in dyslexic children. Sci. Rep..

[13] Thomson J.M., Goswami U. (2010). Learning novel phonological representations in developmental dyslexia: Associations with basic auditory processing of rise time and phonological awareness. Read. Writ..

[14] Ramus F., Marshall C.R., Rosen S., van der Lely H.K. (2013). Phonological deficits in specific language impairment and developmental dyslexia: Towards a multidimensional model. Brain.

[15] Boets B., de Beeck H., Vandermosten M., Scott S.K., Gillebert C.R., Mantini D., Bulthé J., Sunaert S., Wouters J., Ghesquière P. (2013). Intact but less accessible phonetic representations in adults with dyslexia. Science.

[16] Lovio R., Näätänen R., Kujala T. (2010). Abnormal pattern of cortical speech feature discrimination in 6-year-old children at risk for dyslexia. Brain Res..

[17] Nagarajan S., Mahncke H., Salz T., Tallal P., Roberts T., Merzinech M. (1999). Cortical auditory signal processing in poor readers. Proc. Natl. Acad. Sci. USA.

[18] Ziegler J.C., Ferrand L. (1998). Orthography shapes the perception of speech: The consistency effect in auditory word recognition. Psychon. Bull. Rev..

[19] Hämäläinen J.A., Leppänen P.H.T., Guttorm T.K., Lyytinen H. (2007). N1 and P2 components of auditory event-related potentials in children with and without reading disabilities. Clin. Neurophysiol..

[20] Hämäläinen J.A., Leppänen P.H.T., Guttorm T.K., Lyytinen H. (2008). Event-related potentials to pitch and rise time change in children with reading disabilities and typically reading children. Clin. Neurophysiol..

[21] Chobert J., François C., Habib M., Besson M. (2012). Deficit in the preattentive processing of syllables in children with dyslexia. Neuropsychologia.

[22] Näätänen R., Gaillard A.W.K., Mäntysalo S. (1978). Early selective-attention effect on evoked potential reinterpreted. Acta Psychol..

[23] Näätänen R., Paavilainen P., Rinne T., Alho K. (2007). The mismatch negativity (MMN) in basic research of central auditory processing: A review. Clin. Neurophysiol..

[24] Kujala T., Tervaniemi M., Schröger E. (2007). The mismatch negativity in cognitive and clinical neuroscience: Theoretical and methodological considerations. Biol. Psychol..

[25] Näätänen R., Pakarinen S., Rinne T., Takegata R. (2004). The mismatch negativity (MMN): Towards the optimal paradigm. Clin. Neurophysiol..

[26] Lisker L., Abramson A.S. (1967). Some effects of context on voice onset time in English stops. Lang. Speech.

[27] Serniclaes W. (1987). Etude Expérimentale de la Perception du Trait de Voisement des Occlusives du Français. Unpublished Doctoral’s Thesis.

[28] Banai K., Hornickel J., Skoe E., Nicol T., Zecker S.G., Kraus N. (2009). Reading and subcortical auditory function. Cereb. Cortex.

[29] Hämäläinen J., Salminen H., Leppanen P. (2013). Basic auditory processing deficits in dyslexia; A systematic review of the behavioural and event-related potential field evidence. J. Learn. Disabil..

[30] Lovio R., Pakarinen S., Huotilainen M., Alku P., Silvennoinen S., Näätänen R., Kujala T. (2009). Auditory discrimination profiles of speech sound changes in 6-year-old children as determined with the multi-feature MMN paradigm. Clin. Neurophysiol..

[31] Baldeweg T., Richardson A., Watkins S., Foale C., Gruzelier J. (1999). Impaired auditory frequency discrimination in dyslexia detected with mismatch evoked potentials. Ann. Neurol..

[32] Santos A., Joly-Pottuz B., Moreno S., Habib M., Besson M. (2007). Behavioral and event-related potentials evidence for pitch discrimination deficits in dyslexic children: Improvement after intensive phonic intervention. Neuropsychologia.

[33] Cutini S., Szucs D., Mead N., Huss M., Goswami U. (2016). Atypical right hemisphere response to slow temporal modulations in children with developmental dyslexia. Neuroimage.

[34] Power A.J., Colling L.J., Mead N., Barnes L., Goswami U. (2016). Neural encoding of the speech envelope by children with developmental dyslexia. Brain Lang..

[35] Goswami U. (2011). A temporal sampling framework for developmental dyslexia. Trends Cogn. Sci..

[36] Goswami U., Power A.J., Lallier M., Facoetti A. (2014). Oscillatory “temporal sampling” and developmental dyslexia: Toward an over-arching theoretical framework. Front. Hum. Neurosci..

[37] Hämäläinen J., Rupp A., Soltész F., Szücs D., Goswami U. (2012). Reduced phase locking to slow amplitude modulation in adults with dyslexia: An MEG study. NeuroImage.

[38] Lehongre K., Ramus F., Villiermet N., Schwartz D., Giraud A.L. (2011). Altered low-gamma sampling in auditory cortex accounts for the three main facets of dyslexia. Neuron.

[39] Cantiania C., Ortiz-Mantillab S., Rivaa V., Piazzac C., Bettonia R., Musacchia G., Moltenia M., Marino C., Benasich A.A. (2019). Reduced left-lateralized pattern of event-related EEG oscillations in infants at familial risk for language and learning impairment. Neuroimage Clin..

[40] Halliday L.F., Barry J.G., Hardiman M.J., Bishop D.V.M. (2014). Late, not early mismatch responses to changes in frequency are reduced or deviant in children with dyslexia: An event-related potential study. J. Neurodev. Disord..

[41] Bishop D.V.M. (2007). Using mismatch negativity to study central auditory processing in developmental language and literacy impairments: Where are we, and where should we be going?. Psychol. Bull..

[42] Goswami U., Huss M., Mead N., Fosker T., Verney J.P. (2013). Perception of patterns of musical beat distribution in phonological developmental dyslexia: Significant longitudinal relations with word reading and reading comprehension. Cortex.

[43] Huss M., Verney J.P., Fosker T., Mead N., Goswami U. (2011). Music, rhythm, rise time perception and developmental dyslexia: Perception of musical meter predicts reading and phonology. Cortex.

[44] Forgeard M., Schlaug G., Norton A., Rosam C., Iyengar U., Winner E. (2008). The relation between music and phonological processing innormal-reading children and children with dyslexia. Music Percept. Interdiscip. J..

[45] Frey A., François C., Chobert J., Besson M., Ziegler J. (2018). Behavioral and electrophysiological investigation of speech perception deficits in silence, noise and envelope conditions in developmental dyslexia. Neuropsychologia.

[46] Liberman I.Y., Shankweiler D. (1985). Phonology and the problems of learning to read and write. Remedial Spec. Educ..

[47] Overy K. (2003). Dyslexia and music: From timing deficits to musical intervention. Ann. N. Y. Acad. Sci..

[48] Ziegler J.C., Goswami U. (2005). Reading acquisition, developmental dyslexia, and skilled reading across languages: A psycholinguistic grain size theory. Psychol. Bull..

[49] Abrams D.A., Bhatara A., Ryali S., Balaban E., Levitin D.J., Menon V. (2011). Decoding temporal structure in music and speech relies on shared brain resources but elicits different fine-scale spatial patterns. Cereb. Cortex.

[50] Besson M. (1998). Meaning, structure and time in language and music. Neural substrates of cognitive processes. Special issue in homage to Jean Requin. Curr. Psychol. Cogn..

[51] Besson M., Chobert J., Marie C. (2011). Transfer of training between music and speech: Common processing, attention, and memory. Front. Psychol..

[52] Kraus N., Chandrasekaran B. (2010). Music training for the development of auditory skills. Nat. Rev. Neurosci..

[53] Maess B., Koelsch S., Gunter T.C., Friederici A.D. (2001). Musical syntax is processed in Broca’s area: An MEG study. Nat. Neurosci..

[54] Patel A.D. (2003). Language, music, syntax and the brain. Nat. Neurosci..

[55] Patel A.D. (2008). Music, Language, and the Brain.

[56] Zatorre R.J., Gandour J.T. (2008). Neural specializations for speech and pitch: Moving beyond the dichotomies. Philos. Trans. R. Soc. B Biol. Sci..

[57] Kishon-Rabin L., Amir O., Vexler Y., Zaltz Y. (2001). Pitch discrimination: Are professional musicians better than non-musicians?. J. Basic Clin. Physiol. Pharmacol..

[58] Marie C., Kujala T., Besson M. (2012). Musical and linguistic expertise influence preattentive and attentive processing of non-speech sounds. Cortex.

[59] Spiegel M.F., Watson C.S. (1984). Performance on frequency discrimination tasks by musicians and nonmusicians. J. Acoust. Soc. Am..

[60] Tervaniemi M., Just V., Koelsch S., Widmann A., Schröger E. (2005). Pitch discrimination accuracy in musicians vs. nonmusicians: An event-related potential and behavioral study. Exp. Brain Res..

[61] Kühnis J., Elmer S., Meyer M., Jäncke L. (2013). The encoding of vowels and temporal speech cues in the auditory cortex of professional musicians: An EEG study. Neuropsychologia.

[62] Zuk J., Ozernov-Palchik O., Kim H., Lakshminarayanan K., Gabrieli J.D.E., Tallal P., Gaab N. (2013). Enhanced Syllable Discrimination Thresholds in Musicians. PLoS ONE.

[63] Parbery-Clark A., Tierney A., Strait D.L., Kraus N. (2012). Musicians have fine-tuned neural distinction of speech syllables. Neuroscience.

[64] Besson M., Dittinger E., Barbaroux M. (2018). How music training influences language processing: Evidence against informational encapsulation. L’année Psychol..

[65] Nikjeh D.A., Lister J.J., Frisch S.A. (2009). The relationship between pitch discrimination and vocal production: Comparison of vocal and instrumental musicians. J. Acoust. Soc. Am..

[66] Tervaniemi M., Rytkönen M., Schröger E., Ilmoniemi R.J., Näätänen R. (2001). Superior formation of cortical memory traces for melodic patterns in musicians. Learn. Mem..

[67] Milovanov R., Huotilainen M., Esquef P.A.A., Välimäki V., Alku P., Tervaniemi M. (2009). The role of musical aptitude and language skills in preattentive duration determination in school-aged children. Neurosci. Lett..

[68] Chobert J., Marie C., François C., Schön D., Besson M. (2011). Enhanced passive and active processing of syllables in musician children. J. Cogn. Neurosci..

[69] Anvari S.H., Trainor L.J., Woodside J., Levy B.A. (2002). Relation among musical skills, phonological processing and early reading ability in preschool children. J. Exp. Psychol..

[70] Degé F., Schwarzer G. (2011). The effect of a music program on phonological awareness in preschoolers. Front. Psychol..

[71] Slevc L.R., Miyake A. (2006). Individual differences in second language proficiency: Does musical ability matter?. Psychol. Sci..

[72] Moreno S., Marques C., Santos A., Santos M., Castro S.L., Besson M. (2009). Musical training influences linguistic abilities in 8-year-old children: More evidence for brain plasticity. Cereb. Cortex.

[73] Schellenberg E.G. (2004). Music lessons enhance IQ. Psychol. Sci..

[74] Chobert J., François C., Velay J.-L., Besson M. (2014). Twelve months of active musical training in 8 to 10 year old children enhances the preattentive processing of syllabic duration and Voice Onset Time. Cereb. Cortex.

[75] Rugg M.D., Coles M.G.H. (1995). Electrophysiology of Mind.

[76] Donchin E., Ritter W., McCallum C., Callaway E., Tueting P., Koslow S.H. (1978). Cognitive psychophysiology: The endogenous components of the ERP. Event-Related Brain Potentials in Man.

[77] Ritter W., Simon R., Vaughan H.G. (1983). Event-related potential correlates to two stages of information processing in physical and semantic discrimination tasks. Psychophysiology.

[78] Ceponiene R., Shestakova A., Balan P., Alku P., Yiaguchi K., Näätänen R. (2001). Children’s auditory event-related potentials index sound complexity and “speechness”. Int. J. Neurosci..

[79] Lefavrais J. (2005). Test de l’Alouette, rev. version.

[80] Bertrand D., Fluss J., Billard C., Ziegler J.C. (2010). Efficacité, sensibilité, spécificité: Comparaison de différents tests de lecture [Efficiency, sensitivity, specificity: Comparison of different reading tests]. Année Psychol..

[81] Wechsler D. (2003). Wechsler Intelligence Scale for Children.

[82] Korkman M., Kemp S.L., Kirk U. (2004). NEPSY: Bilan Neuropsychologique de L’enfant.

[83] Raven J.C. (1976). Standard Progressive Matrices: Sets A, B, C, D, E.

[84] Chavez M., Day R., Deyell S., Ellis P., Fazio S., Green P., Johnston D., Jonasson B., Levine J., Orler T. (2003). Adobe Audition Software. http://www.adobe.com/products/audition.html.

[85] Boersma P., Weenink D. Praat [Computer Software], Version 4.0. http://www.fon.hum.uva.nl/praat/.

[86] Jasper H.A. (1958). The ten—twenty system of the International Federation. Electroencephologr. Clin. Neurophysiol..

[87] Schröger E., Wolff C. (1998). Attentional orienting and reorienting is indicated by human event-related brain potentials. Neuroreport.

[88] Dufor O., Serniclaes W., Sprenger-Charolles L., Démonet J.F. (2009). Left premotor cortex and allophonic speech perception in dyslexia: A PET study. Neuroimage.

[89] Hoonhorst I., Medina V., Colin C., Markessis E., Radeau M., Deltenre P., Serniclaes W. (2011). Categorical perception of voicing, colors and facial expressions: A developmental study. Speech Commun..

[90] Noordenbos M.W., Segers E., Serniclaes W., Mitterer H., Verhoeven L. (2012). Neural evidence of allophonic perception in children at risk for dyslexia. Neuropsychologia.

[91] Serniclaes W., Heghe S.V., Mousty P., Carré R., Sprenger-Charolles L. (2004). Allophonic mode of speech perception in dyslexia. J. Exp. Child Psychol..

[92] Habib M., Lardy C., Desiles T., Commeiras C., Chobert J., Besson M. (2016). Music and dyslexia: A new musical training method to improve reading and related disorders. Front. Psychol..

[93] Habib M., Commeiras C. (2014). «Mélodys»: Remédiation Cognitivo-Musicale des Troubles de L’apprentissage.

[94] Bidelman G.M., Alain C. (2015). Musical training orchestrates coordinated neuroplasticity in auditory brainstem and cortex to counteract age-related declines in categorical vowel perception. J. Neurosci..

[95] Horvath J., Czigler I., Jacobsen T., Maess B., Schröger E., Winkler I. (2008). MMN or no MMN: No magnitude deviance effect on the MMN amplitude. Psychophysiology.

[96] Näätänen R., Alho K. (1997). Mismatch negativity-the measure for central sound representation accuracy. Audiol. Neurotol..

[97] Marie C., Magne C., Besson M. (2011). Musicians and the metric structure of words. J. Cogn. Neurosci..

[98] Jacquier-Roux M., Valdois S., Zorman M.O. (2005). Outil de Dépistage des Dyslexies.

[99] Maurer U., Bucher K., Brem S., Brandeis D. (2003). Altered responses to tone and phoneme mismatch in kindergartners at familial dyslexia risk. Neuroreport.

[100] Hämäläinen J., Leppänen P.H.T., Torppa M., Müller K., Lyytinen H. (2005). Detection of sound rise time by adults with dyslexia. Brain Lang..

[101] Flaugnacco E., Lopez L., Terribili C., Montico M., Zoia S., Schön D. (2015). Music Training Increases Phonological Awareness and Reading Skills in Developmental Dyslexia: A Randomized Control Trial. PLoS ONE.

[102] Przybylski L., Bedoin N., Krifi-Papoz S., Herbillon V., Roch D., Léculier L., Kotz S.A., Tillmann B. (2013). Rhythmic auditory stimulation influences syntactic processing in children with developmental language disorders. Neuropsychology.

[103] Van der Kleij S.W., Groen M.A., Segers E., Verhoeven L. (2019). Enhanced semantic involvement during word recognition in children with dyslexia. J. Exp. Child Psychol..

[104] Silva P.B., Ueki K., Oliveira D.G., Boggio P.S., Macedo E.C. (2016). Early Stages of Sensory Processing, but Not Semantic Integration, Are Altered in Dyslexic Adults. Front. Psychol..

